# Pancreatic Tuberculosis: Resolution of Pancreatic Pseudocyst and Loculated Ascites Under Tuberculosis Therapy

**DOI:** 10.14309/crj.0000000000001971

**Published:** 2026-01-19

**Authors:** Marie Solange Mukanumviye, Ferehiwot Bekele Getaneh, Samuel Ndagijimana, Antoine Nduwayezu, Vincent Dusabejambo, Eric Rutaganda, Dyna Nyampinga, Zainab Ingabire, Hanna Aberra, Kulwinder S. Dua

**Affiliations:** 1 University Teaching Hospital of Kigali, Kigali, Rwanda; 2 Gitwe District Hospital, Nyanza, Rwanda; 3 King Faisal Hospital, Kigali, Rwanda; 4 University of Rwanda, College of Medicine and Health Sciences, Kigali, Rwanda; 5 Medicine Department at Medical College of Wisconsin, Milwaukee, WI

**Keywords:** pancreatic tuberculosis, extrapulmonary TB, pancreatic pseudocyst, Rwanda, case report, anti-TB therapy

## Abstract

Pancreatic tuberculosis is a rare disease. It mimics both benign and malignant conditions of the pancreas. We describe the case of a man who presented with repetitive epigastric pain and elevated pancreatic enzymes, leading to a presumptive diagnosis of alcohol-induced pancreatitis. Imaging revealed pancreatic pseudocyst, omental thickening, multiple loculated ascites, and bilateral pleural effusions. Despite supportive management for pancreatitis, the patient's symptoms worsened. Further evaluation raised the suspicion of tuberculosis. He was started on antituberculosis therapy. There was complete resolution of his symptoms, pseudocyst and ascites on follow-up imaging. Early suspicion and management of pancreatic tuberculosis may lead to excellent outcomes.

## INTRODUCTION

Tuberculosis (TB) is a common infectious disease, but pancreatic TB is very rare and challenging to diagnose clinically.^1,2^ Although globally TB incidence is reducing, in a Spanish series of 398 patients with TB, 83 patients had extrapulmonary TB. There were 7 (8.4%) patients with intra-abdominal TB but none with pancreatic TB.^3^ Literature on pancreatic TB is primarily from case reports, and the number of reported cases of pancreatic TB is increasing.^4,5^ Its radiological presentation may resemble to acute pancreatitis, chronic pancreatitis, pancreatic abscess, cystic mass lesions of the pancreas, and pancreatic malignancy.^4,6,7^

## CASE REPORT

A 32-year-old man from Rwanda presented with a 2-year history of recurrent severe epigastric pain, poor appetite, and unintentional weight loss of 30 kg. He had no history of TB, HIV, or chronic liver disease. He reported only social alcohol intake. At a primary health facility, he was diagnosed with alcohol-related pancreatitis with a pseudocyst after elevated pancreatic enzymes were noted. Despite alcohol cessation, his symptoms worsened, and he developed progressive abdominal distension.

**Figure 1.**
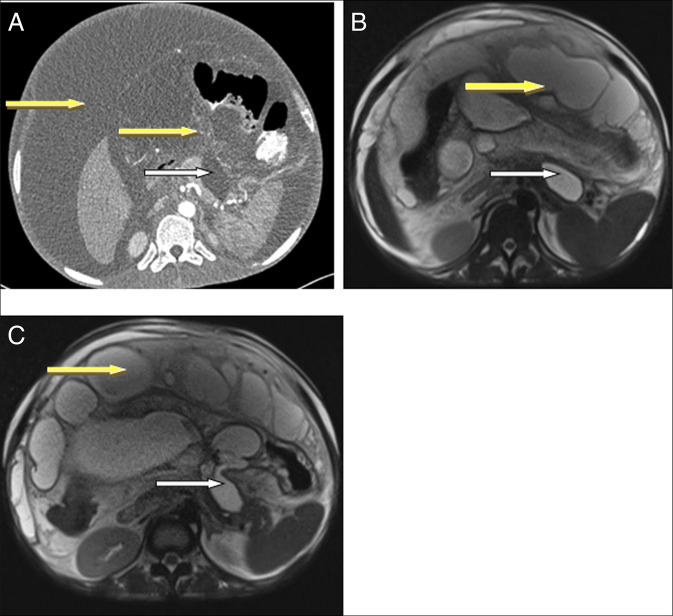
CECT (A) and T2-weighted MRI axial images (B and C) showing cystic pancreatic lesion arising from pancreatic body and tail (white arrows). Omental thickening and multiloculated ascites (yellow arrow). There were no pancreatic ductal dilatation, calcifications, and peripancreatic lymphadenopathy. There were no vascular involvement nor invasion. CECT, contrasted enhanced computed tomography; MRI, magnetic resonance imaging.

On admission to our hospital, he was severely wasted (body mass index of 17.3 kg/m^2^), pale, and wheelchair-bound, with massive ascites and diffuse abdominal tenderness but no organomegaly or stigmata of chronic liver disease. Laboratory tests showed normocytic anemia (Hb 6.5 g/dL) and markedly elevated amylase (3,393 IU/L) and lipase (1,200 IU/L), with otherwise normal biochemistry. carbohydrate antigen 19-9 was not elevated. Ascitic fluid analysis revealed low Serum Ascites Albumin Gradient (0.7 g/dL), elevated amylase, and negative TB-polymerase chain reaction. Abdominal computed tomography (CT) scan demonstrated a pancreatic pseudocyst (homogenous fluid collection with thin pseudo-membrane) involving the body and tail, omental thickening, and multiple loculated ascites (Figure 1). There was no dilatation of the pancreatic duct nor calcifications noted on abdominal magnetic resonance imaging done to exclude pancreatic duct disconnected syndrome and chronic pancreatitis. A chest x-ray was unremarkable. The differential diagnosis at this point were recurrent acute pancreatitis with pseudocyst, autoimmune pancreatitis, cystic pancreatic neoplasms with peritoneal calcinomatosis, chronic pancreatitis, and pancreatic TB with peritoneal involvement.

**Figure 2.**
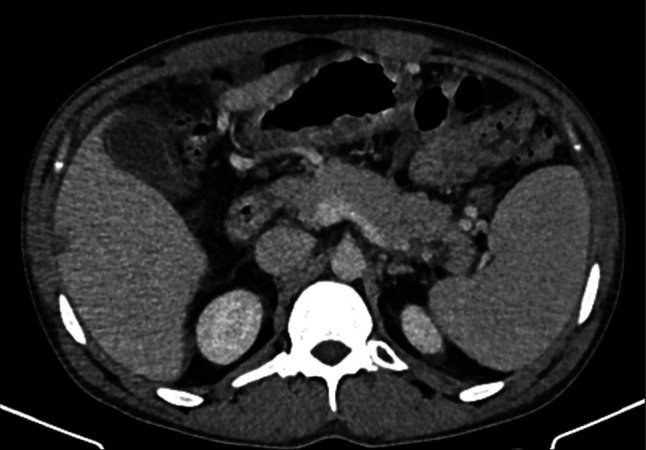
Follow-up CECT 2 years after anti-TB therapy, showing no residual lesions in the pancreas or ascites. CECT, contrasted enhanced computed tomography; TB, tuberculosis.

Given the endemic context (Rwanda, national incidence of 74 per 100,000 people per year), a presumptive diagnosis of pancreatic TB with peritoneal TB was made.^8^ Empirical anti-TB therapy (ATT) resulted in complete clinical and radiologic abnormalities resolution, confirmed on follow-up CT 2 years later (Figure 2). The patient was too sick to tolerate laparoscopic intervention to confirm the diagnosis before initiation of treatment.

Other common etiologies of pancreatitis were systematically excluded. Abdominal imaging showed no gallstones, biliary dilation, or pancreatic ductal abnormalities. Serum calcium and triglyceride levels were normal. There were no radiologic features of chronic pancreatitis such as ductal dilatation or parenchymal calcifications. Alcohol intake was limited and had been discontinued without clinical improvement, making alcohol-related pancreatitis unlikely Table 1.

**Table 1.** Evaluation of common etiologies of pancreatitis in this patient

Etiology	Evaluation	Result
Gallstone pancreatitis	Abdominal ultrasound, MRI of abdomen, abdominal CT scan	No gallstones or biliary dilation
Alcohol-induced pancreatitis	History and abstinence	Limited intake; no improvement after cessation
Hypertriglyceridemia	Serum triglycerides	Normal
Hypercalcemia	Serum calcium	Normal
Post-ERCP pancreatitis	History of a recent performed ERCP	None
Chronic pancreatitis	CT/MRI of abdomen	No ductal dilation or calcifications
Autoimmune pancreatitis	Imaging/clinical features	No suggestive features

CT, computed tomography; ERCP, endoscopic retrograde cholangio-pancreatography; MRI, magnetic resonance imaging.

## DISCUSSION

TB remains a major global health problem, with 10.6 million cases and 1.6 million deaths reported worldwide in 2021 by World Health Organization, making it the leading infectious cause of death. Although pulmonary TB is most common, extrapulmonary TB can affect almost any organ, with lymph node TB being the predominant form after pleural TB. Abdominal TB is the sixth most frequent extrapulmonary manifestation, usually presenting as peritoneal or gastrointestinal disease.^7^ Pancreatic TB is extremely rare, typically occurring between the second and fourth decades of life, with no gender predilection.^7,9^ Our patient was immunocompetent and in his third decade of life, aligning with reported trends.

Pancreatic TB presents with diverse and often nonspecific symptoms. Common features include abdominal pain, poor appetite, weight loss, and occasionally fever or night sweats, although their absence does not rule out the disease, especially in endemic regions. Jaundice may occur with head of pancreas involvement, and complications such as gastrointestinal bleeding can arise from vascular or adjacent organ invasion.^2^ Reported cases range from recurrent pancreatitis initially misdiagnosed as alcohol-related, to asymptomatic presentations discovered incidentally on imaging.^9,10^ Our patient had recurrent abdominal pain, weight loss, ascites, poor appetite, and anemia but denied fever and night sweats.

Pancreatic TB often mimics pancreatic cancer in both clinical and radiological presentations.^2^ Historically, diagnosis was typically made through histological examination of surgical specimen taken from explorative laparotomy. Abdominal ultrasound is the first-line imaging tool but is limited by low sensitivity and operator dependency.^2^ Ultrasound can detect bulky pancreas, altered echogenicity, and enlarged peripancreatic lymph nodes. It can also detect extrapancreatic signs such as ascites, omental thickening, and hepatic or splenic lesions.^2^ Abdominal CT scanning offers greater details and is useful for assessing involvement of surrounding structures. However, CT features are nonspecific and may resemble those of pancreatic tumors.^2^ Radiologically, pancreatic TB most commonly presents as a mass (79%) but can also appear as abscesses, pancreatitis, or pseudocysts.^5^ It may manifest as a complex solid-cystic lesion and occasionally show vascular invasion.^2^

In the reported case, the patient had a pancreatic pseudocyst along with loculated ascites, omental thickening, and pleural effusions. The differential for pancreatic TB include pancreatic malignancy, pancreatic abscess, autoimmune pancreatitis, cystic pancreatic neoplasms, and chronic pancreatitis. In our case reported, TB was suspected based on the constellation of clinical features (significant weight loss, anemia, recurrent pancreatitis), imaging findings (omental thickening, multiloculated ascites, pleural effusions), and epidemiological context (residence in TB-endemic region), despite a negative ascitic fluid TB-polymerase chain reaction and a normal chest X-ray. Tumor markers, mostly carbohydrate antigen 19-9, were not elevated, further lowering suspicion for pancreatic cancer.

Pancreatic TB cannot be confirmed by imaging alone; the preferred diagnostic tool is Endoscopic Ultrasound-Guided Fine Needle Aspiration (EUS-FNA), which allows for detailed visualization and tissue sampling to rule out malignancy and confirm TB.^7^ However, this was not performed in our case due to limited local availability. Histology may reveal granulomatous inflammation, although acid-fast bacilli are rarely seen.^9^ If EUS-FNA is inconclusive or unavailable, laparoscopic or open surgical biopsy may be considered, although our patient was a poor surgical candidate. In endemic regions, when definitive diagnosis is not possible, a therapeutic trial of ATT is often used, with close follow-up to assess response.^7,9^ Our patient was managed with therapeutic trial of ATT, resulting in clinical and radiological improvement by 2 and 6 months, and complete resolution was confirmed on abdominal CT scan at 2 years post-treatment. Pancreatic TB should be considered as differential diagnosis in endemic area, even in immunocompetent patient.

As limitation, our case report relied on response to Empirical ATT, which cannot replace histological confirmation and carries the risk of delayed diagnosis of alternative pathologies, particularly malignancy. We further emphasized that this approach is generally reserved for highly selected cases in TB-endemic settings when tissue diagnosis is not feasible, and that in nonendemic regions, histological confirmation via EUS-FNA is strongly recommended before initiating therapy.

TB remains a common infection globally. Although TB is a commonest infection, pancreatic TB is a rare form, and it can present in a myriad ways. EUS-FNA is the standard diagnostic test as it allows tissue sampling; however, where this is nondiagnostic or not feasible, a trial of empiric ATT can support the diagnosis. Clinicians in high endemic areas should have a high index of suspicion for this disease, particularly in patients with constitutional symptoms and abnormal pancreas on radiological imaging.

## DISCLOSURES

Author contributions: MS Mukanumviye, S. Ndagijimana, A. Nduwayezu, V. Dusabejambo, E. Rutaganda, D. Nyampinga, and H. Aberra contributed to the conceptions, writing, and reviewing of this article. KS. Dua contributed to the review of this article. FB Getaneh and A. Nduwayezu from radiology department contributed to illustration of figures and to the review of this article. M. S. Mukanumviye is the article guarantor.

Acknowledgements: We acknowledge the patient and his family who accepted to share his information to raise awareness of this challenging disease to diagnose.

Financial disclosure: None to report.

Informed consent was obtained for this case report.

## ABBREVIATIONS:

ATT: anti-tuberculosis therapy
BMI: body mass index
CA 19-9: carbohydrate antigen 19-9
CECT: contrasted enhanced computed tomography
CT: computed tomography
EUS-FNA: endoscopic ultrasound–guided fine needle aspiration
Hb: hemoglobin
HIV: human immunodeficiency virus
IU/L: International units per liter
MRI: magnetic resonance imaging
PCR: polymerase chain reaction
SAAG: serum–ascites albumin gradient
WHO: World Health Organization
